# 4,6-Dinitro­benzene-1,3-diamine

**DOI:** 10.1107/S1600536808009318

**Published:** 2008-04-16

**Authors:** Tian Zhou, De-Fu Han, Yong-Jun Hu

**Affiliations:** a School of Chemistry and Life Science, Maoming University, Maoming 525000, People’s Republic of China; b School of Life Science, Changchun Normal University, Changchun 130032, People’s Republic of China

## Abstract

The mol­ecule of the title compound, C_6_H_6_N_4_O_4_, is almost planar, being stabilized by two intra­molecular N—H⋯O hydrogen bonds. Further N—H⋯O links lead to a sheet in the crystal structure.

## Related literature

For related literature, see: Siri & Braunstein (2005 ▶).
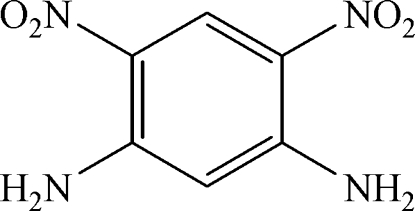


## Experimental

### 

#### Crystal data

C_6_H_6_N_4_O_4_
*M* *_r_* = 198.15
Triclinic, 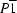

*a* = 7.1294 (6) Å
*b* = 7.1770 (9) Å
*c* = 9.1289 (8) Å
α = 67.710 (6)°
β = 86.692 (6)°
γ = 62.214 (5)°
*V* = 378.30 (7) Å^3^
*Z* = 2
Mo *K*α radiation
μ = 0.15 mm^−1^
*T* = 295 (2) K
0.23 × 0.21 × 0.19 mm

#### Data collection

Bruker APEXII CCD diffractometer
Absorption correction: multi-scan (*SADABS*; Bruker, 2001 ▶) *T*_min_ = 0.967, *T*_max_ = 0.972
2447 measured reflections
1322 independent reflections
1098 reflections with *I* > 2σ(*I*)
*R*_int_ = 0.024

#### Refinement

*R*[*F* ^2^ > 2σ(*F* ^2^)] = 0.051
*wR*(*F* ^2^) = 0.187
*S* = 1.00
1322 reflections
127 parameters
H-atom parameters constrained
Δρ_max_ = 0.50 e Å^−3^
Δρ_min_ = −0.24 e Å^−3^

### 

Data collection: *APEX2* (Bruker, 2004 ▶); cell refinement: *SAINT-Plus* (Bruker, 2001 ▶); data reduction: *SAINT-Plus*; program(s) used to solve structure: *SHELXS97* (Sheldrick, 2008 ▶); program(s) used to refine structure: *SHELXL97* (Sheldrick, 2008 ▶); molecular graphics: *SHELXTL* (Sheldrick, 2008 ▶); software used to prepare material for publication: *SHELXTL*.

## Supplementary Material

Crystal structure: contains datablocks I, global. DOI: 10.1107/S1600536808009318/hb2712sup1.cif

Structure factors: contains datablocks I. DOI: 10.1107/S1600536808009318/hb2712Isup2.hkl

Additional supplementary materials:  crystallographic information; 3D view; checkCIF report

## Figures and Tables

**Table 1:** Hydrogen-bond geometry (Å, °)

*D*—H⋯*A*	*D*—H	H⋯*A*	*D*⋯*A*	*D*—H⋯*A*
N1—H1*A*⋯O3^i^	0.86	2.24	3.074 (2)	162
N1—H1*A*⋯O1^ii^	0.86	2.47	2.917 (2)	113
N1—H1*B*⋯O4	0.86	2.05	2.667 (3)	128
N2—H2*A*⋯O2^i^	0.86	2.31	3.098 (2)	152
N2—H2*B*⋯O1	0.86	2.03	2.642 (2)	128
N2—H2*B*⋯O4^iii^	0.86	2.33	2.964 (3)	131

Symmetry codes: (i) 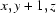; (ii) 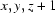; (iii) 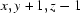.

**Fig. 1.**
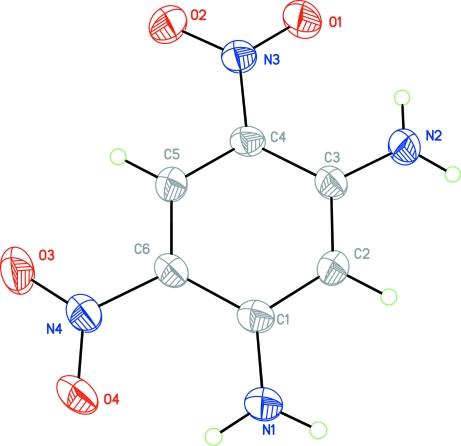
The molecular structure of (I), drawn with 50% displacement ellipsoids for the non-hydrogen atoms. The hydrogen bonds are shown as double-dashed lines.

## supplementary crystallographic information

### Comment

As part of the ongoing investigations of biological structure-property relationships in amino-containing molecules (Siri & Braunstein, 2005), we now report the synthesis and structure of the title compound, (I), (Fig. 1).

The molecule of (I) is almost planar, being stabilised by two intramolecular N-H···O interactions (Table 1).  The aromatic ring makes dihedral angles of 3.7 (2)° and 4.6 (3)° with the N3/O1/O2 and N4/O3/O4 nitro groups, respectively. Further intermolecular N-H···O hydrogen bonds result in (100) sheets in the crystal (Fig. 2).

### Experimental

80 ml concentrated HN0_3_ was added dropwise to 29.2 g 1,3-dichlorobenzene in 150 ml oleum (25% sulfur trioxide) and the mixture was stirred for 30 minutes.  The resulting solution was poured over 2000 g crushed ice. After the ice has melted, sufficient 30% sodium hydroxide solution was added to achieve a pH of 7 and 24.2 g of 1,3-dinitro-4,6-dichlorobenzene (II) was obtained after filtration and drying.   Then, 7.2 g of (II) and 50 ml 30% aqueous ammonia were sealed in a 100-ml autoclave and heated to 443 K for 24 h. After cooling to room temperature, 5.6 g (23% yield) of colourless blocks of (I) were recovered.  Anal. Calc. for C_6_H_6_N_4_O_4_: C 36.34, H 3.03, N 28.28%; Found: C 36.32, H 3.01, N 28.29%.

### Refinement

The H atoms were placed in calculated positions with C—H = 0.93Å and N—H = 0.86Å and refined as riding with *U*_iso_(H) = 1.2*U*_eq_(carrier).

### Crystal data

**Table:**

C_6_H_6_N_4_O_4_	*Z* = 2
*M**_r_* = 198.15	*F*(000) = 204
Triclinic, *P*1	*D*_x_ = 1.740 Mg m^−^^3^
Hall symbol: -P 1	Mo *K*α radiation, λ = 0.71073 Å
*a* = 7.1294 (6) Å	Cell parameters from 1322 reflections
*b* = 7.1770 (9) Å	θ = 3.4–25.1°
*c* = 9.1289 (8) Å	µ = 0.15 mm^−^^1^
α = 67.710 (6)°	*T* = 295 K
β = 86.692 (6)°	Block, colourless
γ = 62.214 (5)°	0.23 × 0.21 × 0.19 mm
*V* = 378.30 (7) Å^3^	

### Data collection

**Table:**

Bruker APEXII CCD diffractometer	1322 independent reflections
Radiation source: fine-focus sealed tube	1098 reflections with *I* > 2σ(*I*)
graphite	*R*_int_ = 0.024
φ and ω scans	θ_max_ = 25.1°, θ_min_ = 3.4°
Absorption correction: multi-scan (*SADABS*; Bruker, 2001)	*h* = −8→8
*T*_min_ = 0.967, *T*_max_ = 0.972	*k* = −3→8
2447 measured reflections	*l* = −9→10

### Refinement

**Table:**

Refinement on *F*^2^	Primary atom site location: structure-invariant direct methods
Least-squares matrix: full	Secondary atom site location: difference Fourier map
*R*[*F*^2^ > 2σ(*F*^2^)] = 0.051	Hydrogen site location: inferred from neighbouring sites
*wR*(*F*^2^) = 0.187	H-atom parameters constrained
*S* = 1.00	*w* = 1/[σ^2^(*F*_o_^2^) + (0.15*P*)^2^ + 0.0584*P*] where *P* = (*F*_o_^2^ + 2*F*_c_^2^)/3
1322 reflections	(Δ/σ)_max_ < 0.001
127 parameters	Δρ_max_ = 0.50 e Å^−^^3^
0 restraints	Δρ_min_ = −0.24 e Å^−^^3^

### Special details

**Table:**

Geometry. All e.s.d.'s (except the e.s.d. in the dihedral angle between two l.s. planes) are estimated using the full covariance matrix. The cell e.s.d.'s are taken into account individually in the estimation of e.s.d.'s in distances, angles and torsion angles; correlations between e.s.d.'s in cell parameters are only used when they are defined by crystal symmetry. An approximate (isotropic) treatment of cell e.s.d.'s is used for estimating e.s.d.'s involving l.s. planes.
Refinement. Refinement of *F*^2^ against ALL reflections. The weighted *R*-factor *wR* and goodness of fit *S* are based on *F*^2^, conventional *R*-factors *R* are based on *F*, with *F* set to zero for negative *F*^2^. The threshold expression of *F*^2^ > σ(*F*^2^) is used only for calculating *R*-factors(gt) *etc*. and is not relevant to the choice of reflections for refinement. *R*-factors based on *F*^2^ are statistically about twice as large as those based on *F*, and *R*- factors based on ALL data will be even larger.

### Fractional atomic coordinates and isotropic or equivalent isotropic displacement parameters (Å^2^)

**Table:**

	*x*	*y*	*z*	*U*_iso_*/*U*_eq_	
C1	0.2508 (3)	0.6981 (4)	0.8212 (2)	0.0307 (5)	
C2	0.2639 (3)	0.8653 (4)	0.6845 (2)	0.0325 (6)	
H2	0.2723	0.9844	0.6965	0.039*	
C3	0.2654 (3)	0.8654 (3)	0.5319 (2)	0.0291 (5)	
C4	0.2464 (3)	0.6832 (3)	0.5162 (2)	0.0284 (5)	
C5	0.2400 (3)	0.5121 (3)	0.6491 (2)	0.0308 (5)	
H5	0.2317	0.3932	0.6369	0.037*	
C6	0.2457 (3)	0.5127 (3)	0.7994 (2)	0.0312 (5)	
N1	0.2441 (3)	0.7171 (3)	0.9615 (2)	0.0399 (6)	
H1A	0.2478	0.8319	0.9678	0.048*	
H1B	0.2360	0.6146	1.0455	0.048*	
N2	0.2856 (3)	1.0298 (3)	0.4091 (2)	0.0397 (5)	
H2A	0.2977	1.1347	0.4248	0.048*	
H2B	0.2865	1.0300	0.3148	0.048*	
N3	0.2326 (3)	0.6701 (3)	0.3643 (2)	0.0332 (5)	
N4	0.2487 (3)	0.3197 (3)	0.9290 (2)	0.0390 (5)	
O1	0.2415 (3)	0.8172 (3)	0.24312 (17)	0.0490 (5)	
O2	0.2106 (3)	0.5129 (3)	0.35769 (19)	0.0501 (5)	
O3	0.2537 (4)	0.1638 (3)	0.9013 (2)	0.0635 (7)	
O4	0.2458 (3)	0.3152 (3)	1.06588 (18)	0.0539 (6)	

### Atomic displacement parameters (Å^2^)

**Table:**

	*U*^11^	*U*^22^	*U*^33^	*U*^12^	*U*^13^	*U*^23^
C1	0.0380 (11)	0.0317 (11)	0.0225 (10)	−0.0169 (9)	0.0053 (8)	−0.0108 (8)
C2	0.0471 (12)	0.0284 (11)	0.0280 (12)	−0.0218 (9)	0.0058 (9)	−0.0126 (9)
C3	0.0360 (11)	0.0269 (11)	0.0236 (10)	−0.0160 (8)	0.0051 (8)	−0.0082 (8)
C4	0.0354 (11)	0.0300 (11)	0.0207 (10)	−0.0149 (9)	0.0052 (7)	−0.0122 (9)
C5	0.0401 (11)	0.0256 (10)	0.0287 (11)	−0.0172 (9)	0.0043 (8)	−0.0112 (9)
C6	0.0415 (11)	0.0288 (11)	0.0220 (11)	−0.0186 (9)	0.0041 (8)	−0.0065 (9)
N1	0.0684 (13)	0.0374 (11)	0.0218 (10)	−0.0307 (10)	0.0093 (8)	−0.0132 (8)
N2	0.0674 (13)	0.0363 (10)	0.0241 (9)	−0.0343 (10)	0.0092 (8)	−0.0091 (8)
N3	0.0443 (10)	0.0319 (10)	0.0253 (9)	−0.0185 (8)	0.0041 (7)	−0.0129 (8)
N4	0.0593 (12)	0.0337 (11)	0.0265 (9)	−0.0268 (9)	0.0078 (8)	−0.0090 (8)
O1	0.0861 (13)	0.0490 (11)	0.0202 (8)	−0.0406 (9)	0.0121 (7)	−0.0121 (8)
O2	0.0847 (12)	0.0457 (10)	0.0358 (10)	−0.0388 (9)	0.0054 (8)	−0.0217 (8)
O3	0.1255 (18)	0.0439 (11)	0.0397 (10)	−0.0569 (12)	0.0171 (10)	−0.0147 (9)
O4	0.0979 (14)	0.0498 (11)	0.0214 (9)	−0.0460 (10)	0.0134 (8)	−0.0087 (8)

### Geometric parameters (Å, °)

**Table:**

C1—N1	1.334 (3)	C5—H5	0.9300
C1—C2	1.401 (3)	C6—N4	1.432 (3)
C1—C6	1.435 (3)	N1—H1A	0.8600
C2—C3	1.392 (3)	N1—H1B	0.8600
C2—H2	0.9300	N2—H2A	0.8600
C3—N2	1.344 (3)	N2—H2B	0.8600
C3—C4	1.434 (3)	N3—O1	1.229 (2)
C4—C5	1.377 (3)	N3—O2	1.233 (2)
C4—N3	1.437 (3)	N4—O3	1.222 (3)
C5—C6	1.377 (3)	N4—O4	1.236 (3)
			
N1—C1—C2	119.87 (19)	C5—C6—N4	116.47 (18)
N1—C1—C6	123.84 (19)	C5—C6—C1	120.45 (19)
C2—C1—C6	116.29 (19)	N4—C6—C1	123.07 (19)
C3—C2—C1	124.43 (19)	C1—N1—H1A	120.0
C3—C2—H2	117.8	C1—N1—H1B	120.0
C1—C2—H2	117.8	H1A—N1—H1B	120.0
N2—C3—C2	119.92 (18)	C3—N2—H2A	120.0
N2—C3—C4	123.51 (19)	C3—N2—H2B	120.0
C2—C3—C4	116.57 (18)	H2A—N2—H2B	120.0
C5—C4—C3	120.39 (19)	O1—N3—O2	121.21 (18)
C5—C4—N3	117.18 (18)	O1—N3—C4	119.26 (17)
C3—C4—N3	122.43 (19)	O2—N3—C4	119.52 (17)
C4—C5—C6	121.69 (19)	O3—N4—O4	121.72 (17)
C4—C5—H5	119.2	O3—N4—C6	119.01 (18)
C6—C5—H5	119.2	O4—N4—C6	119.27 (18)

### Hydrogen-bond geometry (Å, °)

**Table:**

*D*—H···*A*	*D*—H	H···*A*	*D*···*A*	*D*—H···*A*
N1—H1A···O3^i^	0.86	2.24	3.074 (2)	162
N1—H1A···O1^ii^	0.86	2.47	2.917 (2)	113
N1—H1B···O4	0.86	2.05	2.667 (3)	128
N2—H2A···O2^i^	0.86	2.31	3.098 (2)	152
N2—H2B···O1	0.86	2.03	2.642 (2)	128
N2—H2B···O4^iii^	0.86	2.33	2.964 (3)	131

Symmetry codes: (i) *x*, *y*+1, *z*; (ii) *x*, *y*, *z*+1; (iii) *x*, *y*+1, *z*−1.

## References

[bb1] Bruker (2001). *APEX2* Bruker AXS Inc., Madison, Wisconsin, USA.

[bb2] Bruker (2004). *SAINT-Plus* and *SADABS* Bruker AXS Inc., Madison, Wisconsin, USA.

[bb3] Sheldrick, G. M. (2008). *Acta Cryst.* A**64**, 112–122. 10.1107/S0108767307043930 18156677

[bb4] Siri, O. & Braunstein, P. (2005). *New J. Chem* **29**, 75–78.

